# Identification of Nedd9 as a TGF-β-Smad2/3 Target Gene Involved in RANKL-Induced Osteoclastogenesis by Comprehensive Analysis

**DOI:** 10.1371/journal.pone.0157992

**Published:** 2016-06-23

**Authors:** Yasunori Omata, Shinya Nakamura, Takuma Koyama, Tetsuro Yasui, Jun Hirose, Naohiro Izawa, Takumi Matsumoto, Yuuki Imai, Sachiko Seo, Mineo Kurokawa, Shuichi Tsutsumi, Yuho Kadono, Chikao Morimoto, Hiroyuki Aburatani, Takeshi Miyamoto, Sakae Tanaka

**Affiliations:** 1 Department of Orthopaedic Surgery, Faculty of Medicine, The University of Tokyo, Bunkyo-ku, Tokyo, Japan; 2 Division of Integrative Pathophysiology, Proteo-Science Center, Graduate School of Medicine, Ehime University, Ehime 791–0295, Japan; 3 Department of Hematology and Oncology, Graduate School of Medicine, University of Tokyo, Tokyo, Japan; 4 Genome Science Division, Research Center for Advanced Science and Technology (RCAST), The University of Tokyo, Tokyo, Japan; 5 Department of Therapy Development and Innovation for Immune Disorders and Cancers, Juntendo University, Tokyo, Japan; 6 Department of Orthopedic Surgery, Keio University, Tokyo, Japan; Faculté de médecine de Nantes, FRANCE

## Abstract

TGF-ß is a multifunctional cytokine that is involved in cell proliferation, differentiation and function. We previously reported an essential role of the TGF-ß -Smad2/3 pathways in RANKL-induced osteoclastogenesis. Using chromatin immunoprecipitation followed by sequencing, we comprehensively identified Smad2/3 target genes in bone marrow macrophages. These genes were enriched in the gene population upregulated by TGF-ß and downregulated by RANKL. Recent studies have revealed that histone modifications, such as trimethylation of histone H3 lysine 4 (H3K4me3) and lysine 27 (H3K27me3), critically regulate key developmental steps. We identified *Nedd9* as a Smad2/3 target gene whose histone modification pattern was converted from H3K4me3(+)/H3K4me27(+) to H3K4me3(+)/H3K4me27(-) by TGF-ß. *Nedd9* expression was increased by TGF-ß and suppressed by RANKL. Overexpression of *Nedd9* partially rescued an inhibitory effect of a TGF-ß inhibitor, while gene silencing of *Nedd9* suppressed RANKL-induced osteoclastogenesis. RANKL-induced osteoclastogenesis were reduced and stimulatory effects of TGF-ß on RANKL-induced osteoclastogenesis were partially abrogated in cells from *Nedd9*-deficient mice although knockout mice did not show abnormal skeletal phenotypes. These results suggest that *Nedd9* is a Smad2/3 target gene implicated in RANKL-induced osteoclastogenesis.

## Introduction

Skeletal homeostasis is strictly controlled by osteoclasts, which mediate bone resorption, and osteoblasts, which regulate bone formation. Osteoclasts are multinucleated cells derived from monocyte-macrophage lineage hematopoietic progenitor cells and specifically differentiated for bone resorption [1]. The differentiation of osteoclasts is regulated by two cytokines: receptor activator of nuclear factor kappa B ligand (RANKL) and macrophage colony-stimulating factor (M-CSF). In addition to these two essential cytokines, we recently reported a critical role for TGF-ß in osteoclastogenesis [2] [3]. TGF-ß is abundantly stored in bone matrix and has profound biological functions such as angiogenesis, cellular differentiation, apoptosis and bone homeostasis [4] [5]. The binding of TGF-ß to its type II receptors recruits and phosphorylates type I receptors, which in turn activate downstream signaling including Smad and non-Smad pathways [6]. Phosphorylated Smad2/3 forms a complex with Smad4, and the molecular complex translocates into the nucleus and regulates specific gene expression [7] [8] [9].

We previously reported that TGF-ß is required for osteoclast differentiation in response to RANKL and M-CSF by regulating the interaction of Smad2/3 with TRAF (tumor necrosis factor receptor-associated factor) 6, an adaptor molecule associated with RANK [2]. In addition, we identified Smad2/3-binding sites in open chromatin regions during osteoclastogenesis and found that Smad2/3 binding is necessary for the nuclear translocation of c-Fos, an essential transcription factor for osteoclastogenesis [3]. Moreover, it was reported that combined treatment of TGF-ß and TNF-α promotes maximal osteoclast formation compared to treatment with other cytokine combinations in the presence of RANKL based on a multiparameter cytokine assay [10]. However, direct target genes that regulate osteoclast differentiation downstream of TGF-ß-Smad2/3 pathways still remain elusive.

Multiple epigenetic modifications, such as DNA methylation and, histone acetylation and methylation, are involved in organization of chromatin structures at various levels and regulation of gene expression. The methylated sites in H3 or H4 are mainly located in the histone tail (H3K4, H3K9, H3K36 and H4K20) and the center of the nucleosome (H3K79) [11]. Among the five histones, which are designated as H1, H2A, H2B, H3 and H4 [12], Stahl et al. reported that the methylation of histone H3 at lysine 4 is highly conserved and correlated with transcriptionally active nuclei in *Tetrahymena* [13]. Bernstein et al. revealed that histone modifications such as trimethylation of histone H3 lysine 4 (H3K4me3) and lysine 27 (H3K27me3) play a critical role in gene expression, and in embryonic stem cells, key developmental genes tend to change histone modification patterns from the H3K4me3/H3K27me3 bivalent pattern to the H3K4me3 monovalent pattern [14]. Similar modifications of histone methylation have been observed in many other types of cells, and we previously reported that RANKL induced bivalent to monovalent changes in the *nuclear factor of activated T-cells cytoplasmic 1* (*NFATc1*) gene during osteoclastogenesis [15]. Through chromatin immunoprecipitation with sequencing (ChIP-seq) analysis using anti-Smad2/3, anti-H3K4me3 and anti-H3K27me3 antibodies [16], here we investigated Smad2/3-regulating genes that are critically involved in the differentiation of osteoclasts and identified Nedd9 as a putative regulator of osteoclastogenesis downstream of TGF-ß-Smad2/3 pathways.

## Results

### Identification of genes regulated by the TGF-β-Smad2/3 axis

To identify genes regulated by the TGF-ß-Smad2/3 axis, we performed ChIP-seq analysis using anti-Smad2/3 antibody in M-CSF-dependent bone marrow macrophages (BMMs) treated with 2 ng/ml TGF-ß for 1.5 h. Total read number was 15,108,905, and 10,837,516 reads (71.7%) were mapped to the mouse genome. A total of 2,786 Smad2/3-binding regions (SBRs) were identified (peak signal ratio ≥8). Genes with peak positions of SBRs between 10 kb upstream from transcription start sites (TSSs) and first intron were defined as Smad2/3 target genes, and 903 genes were selected as Smad2/3 target genes.

**Fig 1:**
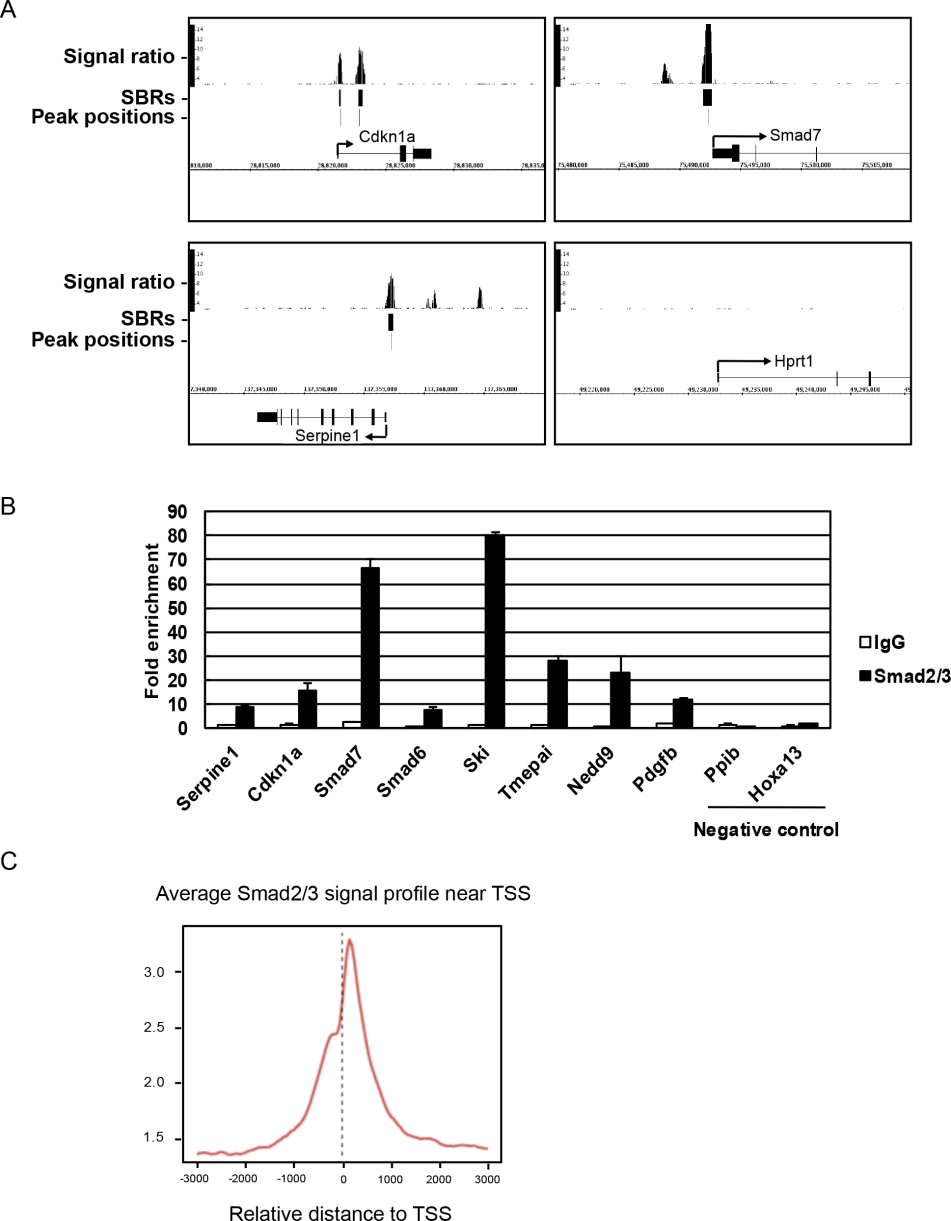
Identification of Smad2/3 binding sites. (A) BMMs were treated with 2 ng/ml TGF-ß for 1.5 h and cells were subjected to ChIP-seq analysis using anti-Smad2/3 antibody. Three known TGF-ß target genes (*Cdkn1a*, *Serpine1*, *and Smad7*) and a negative control gene (*Hprt1*) were analyzed as representative examples. Smad2/3-binding regions (SBRs; peak signal ratio ≥8) and the peak position of each SBR are shown by black bars. (B) Eight positive regions and two negative regions for Smad2/3 binding were selected from ChIP-seq data and validated by realtime PCR. ChIP using mouse IgG was used as control. Values are presented as n-fold enrichment over Hprt1. (C) Average Smad2/3 signal profile around transcriptional start site (TSS) in ChIP-seq analysis. Smad2/3 binding was enriched around TSS.

As shown in Fig 1A, we confirmed that Smad2/3 bound to the promoter regions of known TGF-ß-regulating genes such as *Cdkn1a*, *Smad7* and *Serpine1*. We selected Smad2/3-binding regions in 8 of these genes (*Serpine1*, *Cdkn1a*, *Smad7*, *Smad6*, *Ski*, *Tmepai*, *Nedd9 and Pdgfb*) and confirmed a TGF-ß-dependent increase in Smad2/3 binding by ChIP-realtime PCR (Fig 1B). SBRs were widely distributed from distant upstream regions to intronic regions, with prominent enrichment close to the TSSs of RefSeq genes (Fig 1C).

### Histone modification by TGF-β

**Fig 2:**
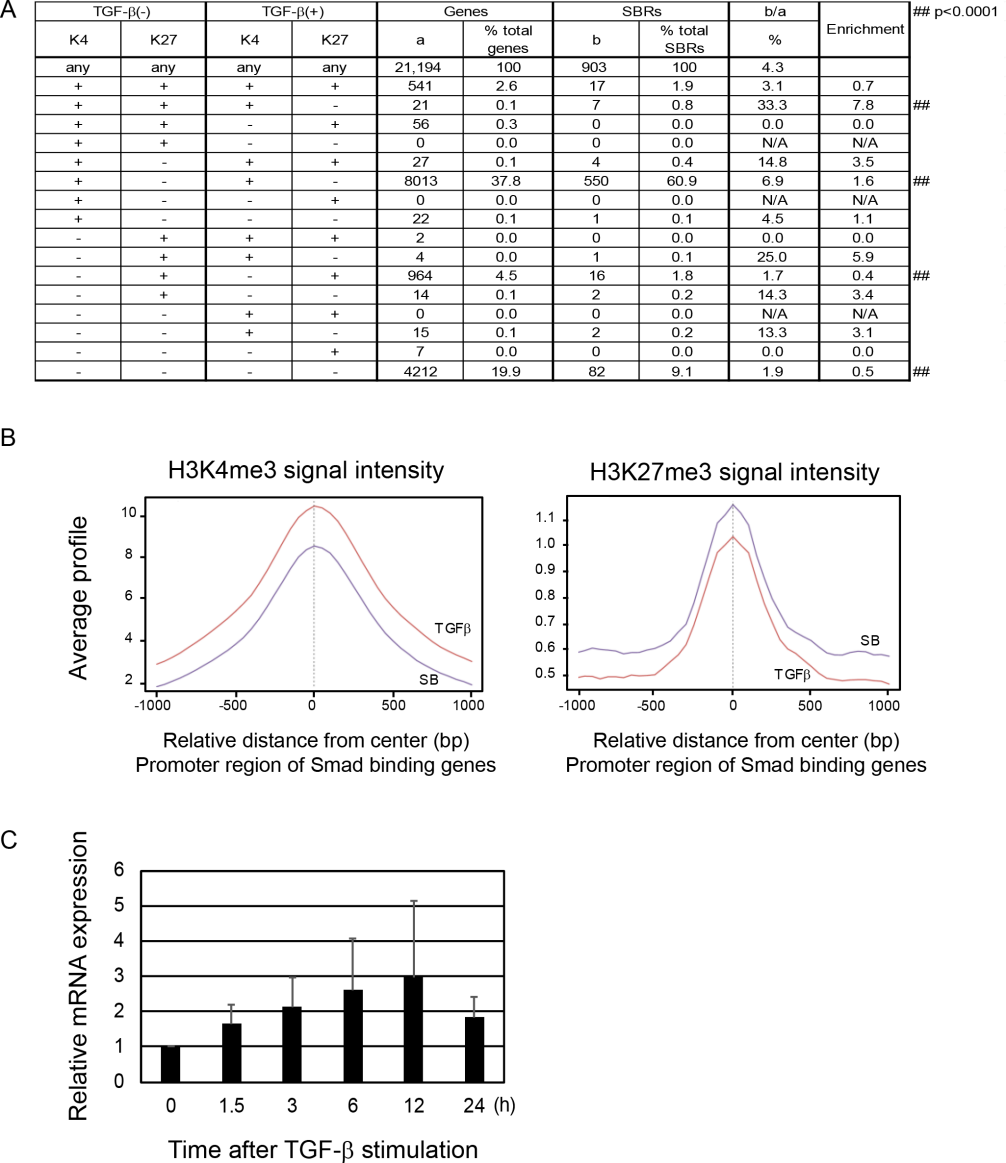
(A) Genes with H3K4me3 peaks within +/- 1 kb from TSS were defined as K4(+) genes, and genes with H3K27me3 peaks within +/- 1 kb from TSS were defined as K27(+) genes. Genes with each combination of K4 and K27 status were identified and enrichment of Smad2/3 target genes was calculated. Highest enrichment was observed in genes with K4(+)K27(+) marks in TGF-ß(-) BMMs and K4(+)K27(-) marks in TGF-ß(+) BMM. (B) The intensity of histone marks around TSS of Smad2/3 target genes. The signal intensity of H3K4me3 in BMMs treated with TGF-ß was higher than those treated with SB431542, while the signal intensity of H3K4me27 was lower in TGF-ß(+) BMMs than that in TGF-ß(-) BMMs. (C) mRNA expression of Smad target genes with K4(+)K27(+) marks in TGF-ß(-) BMMs and K4(+)K27(-) marks in TGF-ß(+) BMM.

To analyze TGF-ß-induced histone modification, BMMs treated with 1 ng/ml TGF-ß [designated as TGF-ß(+) BMMs] or 5 μM SB431542 [TGF-ß(-) BMMs] were subjected to ChIP-seq analysis using anti-H3K4me3 or anti-H3K27me3 antibody. SB431542 is a potent and specific kinase inhibitor of TGF-ß type I receptor that strongly suppresses RANKL-induced osteoclastogenesis [2]. Genes with H3K4me3 peaks within +/- 1kb from the TSSs were defined as K4(+) genes (peak signal ratio ≥8), and genes with H3K27me3 peaks within +/- 1kb from the TSSs were defined as K27(+) genes (peak signal ratio ≥5). A total of 9,962 and 9,505 K4(+) genes and 2,600 and 2,827 K27(+) genes were identified in TGF-ß(-) and TGF-ß(+) BMMs, respectively. Smad2/3 target genes were significantly enriched in genes with K4(+)K27(+) marks in TGF-ß(-) BMMs and K4(+)K27(-) marks in TGF-ß(+) (7.8 fold enrichment; *P* <10^−5^ by chi square test) (Fig 2A). The average signal intensity of H3K4me3 around TSS was higher in TGF-ß(+) BMMs than in TGF-ß(-) BMMs while that of H3K27me3 was lower (Fig 2B). Indeed, mRNA expression of Smad target genes with K4(+)K27(+) marks in TGF-ß(-) BMMs and K4(+)K27(-) marks in TGF-ß(+) BMM were up-regulated after TGF-ß stimulation (Fig 2C).

### TGF-β positively and RANKL negatively regulates Smad2/3 target genes

**Fig 3:**
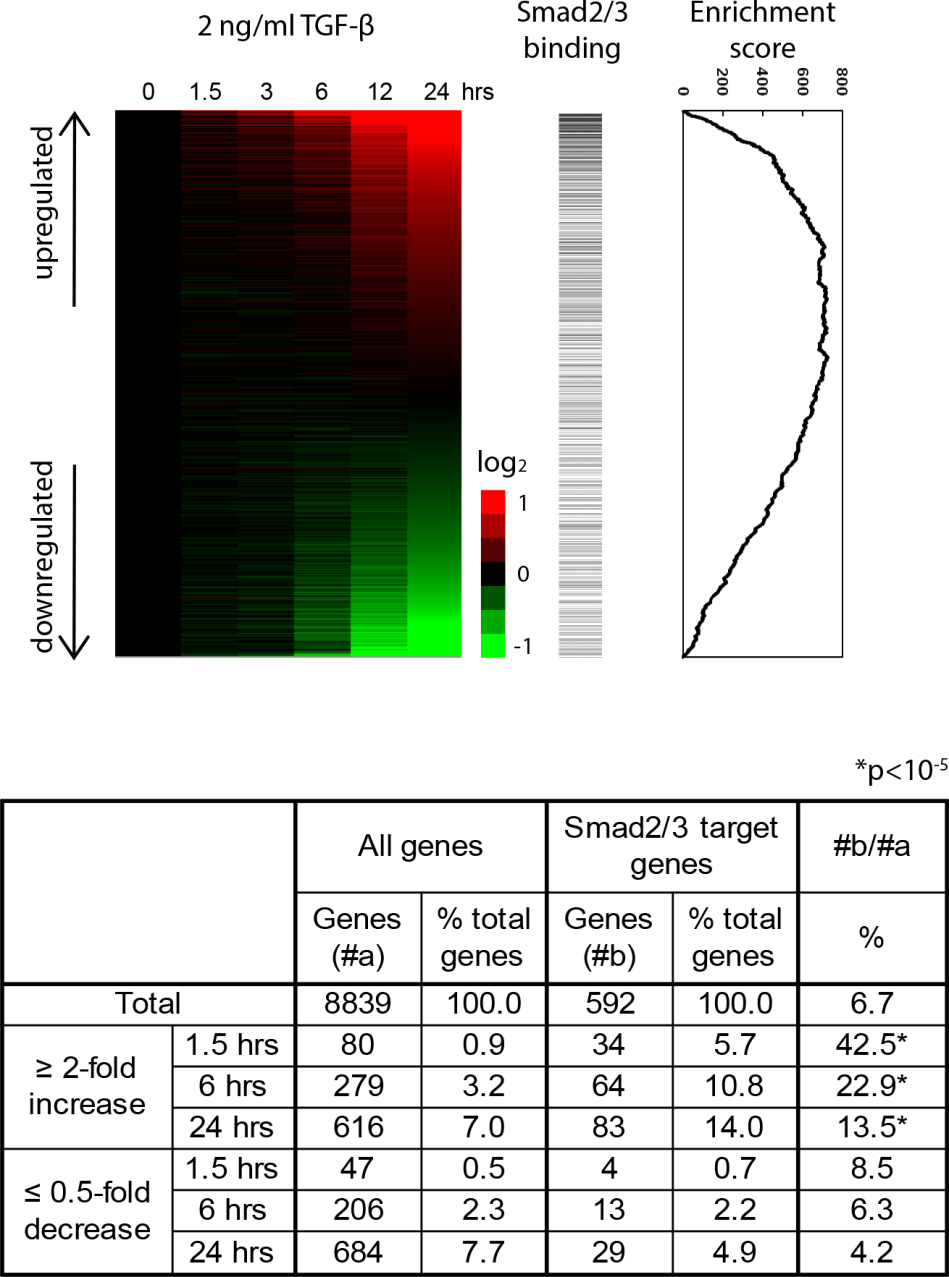
Identification of downstream effectors of TGF-ß in osteoclastogenesis. Upper: The expression scores after TGF-ß stimulation of BMMs relative to time 0 are illustrated by a heat map with red or green color representing increased or decreased gene expression, respectively. The probes were sorted by the ratio at 24 h (ranked gene list). Horizontal bars indicate Smad2/3 target genes. GSEA enrichment scores are graphically shown in the right panel [17]. Smad2/3 target genes were significantly enriched in genes upregulated by TGF-ß. Lower: The expression scores after RANKL stimulation of BMMs relative to time 0 are illustrated by a heat map. Genes whose expression was upregulated more than 2-fold or downregulated less than 0.5-fold relative to RANKL treatment were counted. Enrichment of Smad2/3 target genes in up- or downregulated genes was evaluated by chi-square test (*P* < 10^−5^).

Using 14,177 probes (8,839 genes) with expression values of more than 70 by MOE430 GeneChips at least one time point, we found that Smad2/3 target genes were significantly enriched in the genes whose expression was more than 2-fold upregulated, but not in those whose expression was less than 0.5-fold downregulated, by TGF-ß (Fig 3, upper and lower panels; *P* < 10^−5^ by chi square test). Enrichment scores calculated by Gene Set Enrichment Analysis (GSEA) [17] exhibited statistically significant enrichment (*P* < 10^−6^) (Fig 3, lower panel).

**Fig 4:**
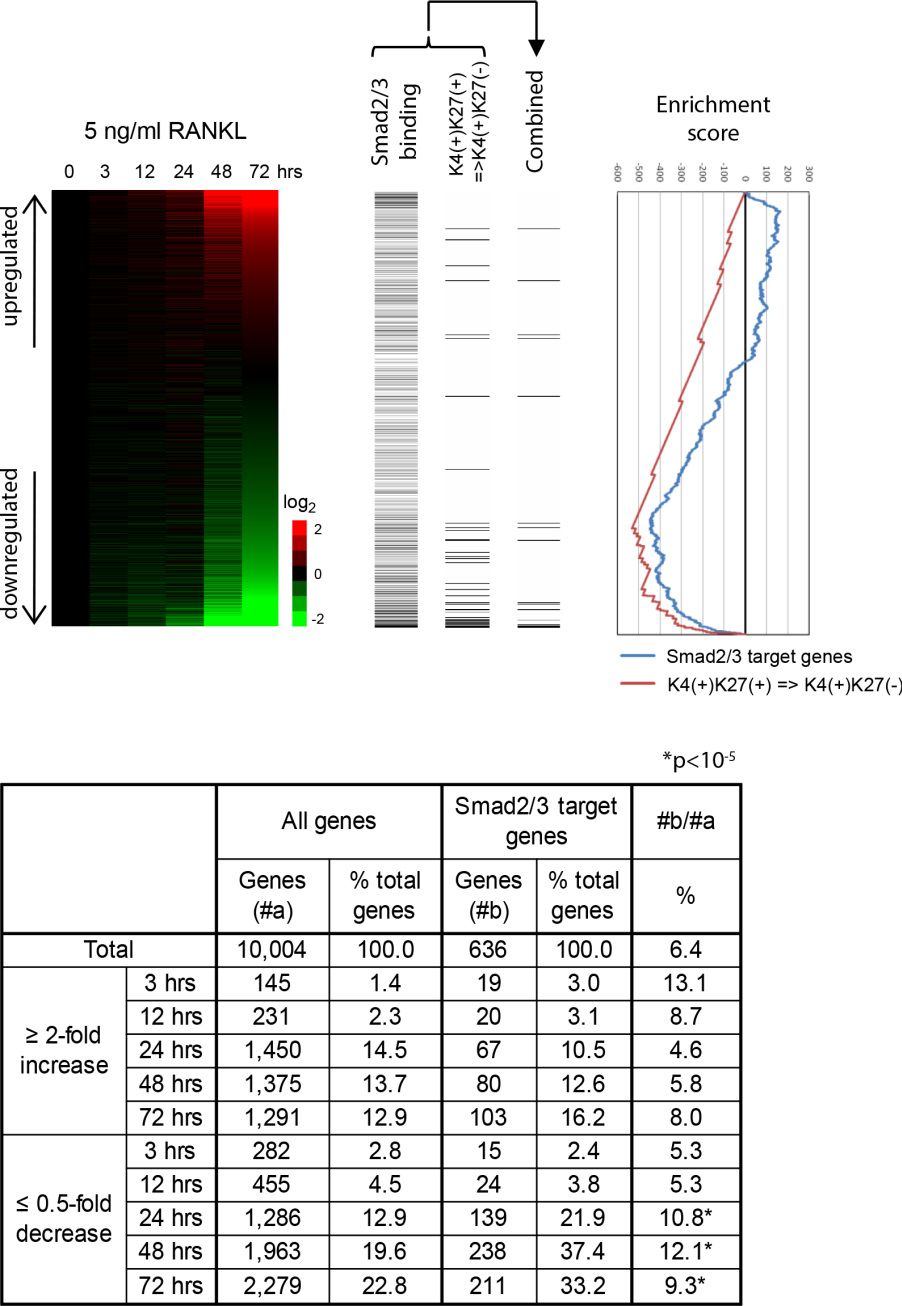
Upper: TGF-ß(+) BMMs were treated with 5 nM GST-RANKL for 0, 3, 12, 24, 48, and 72 h. Relative expression levels of Smad2/3 target genes as compared with those in time 0 are illustrated by a heat map. The probes were sorted by the value at 72 h after RANKL treatment. Horizontal bars indicate Smad2/3 target genes and genes with K4(+)K27(+) marks in TGF-ß(-) BMMs and K4(+)K27(-) marks in TGF-ß(+) BMMs. GSEA enrichment scores are graphically shown in the right panel. Lower: Enrichment of Smad2/3 target genes in the gene population whose relative expression levels were upregulated more than 2-fold or downregulated less than 0.5-fold by RANKL. Smad2/3 target genes were significantly enriched in downregulated genes by RANKL.

We then analyzed the change of the expression of Smad2/3 target genes by RANKL stimulation. We used 16,631 probes (10,004 genes) with expression values of more than 70 at least one time point for further analysis. Interestingly, Smad2/3 target genes were significantly enriched in the genes whose expression was less than 0.5-fold downregulated, but not in those whose expression was more than 2-fold upregulated, after 24, 48 and 72 h of RANKL stimulation (Fig 4, upper and lower panels; *P* < 10^−5^ by chi square test). In addition, genes whose histone modification was changed from K4(+)K27(+) to K4(+)K27(-) patterns by TGF-ß treatment were enriched in downregulated genes by RANKL (Fig 4, upper panel). The statistical significance of the enrichment was analyzed by calculating GSEA enrichment scores (*P* < 10^−6^) (Fig 4, lower panel).

### Identification of Nedd9 as a possible Smad2/3 target in BMMs

**Fig 5:**
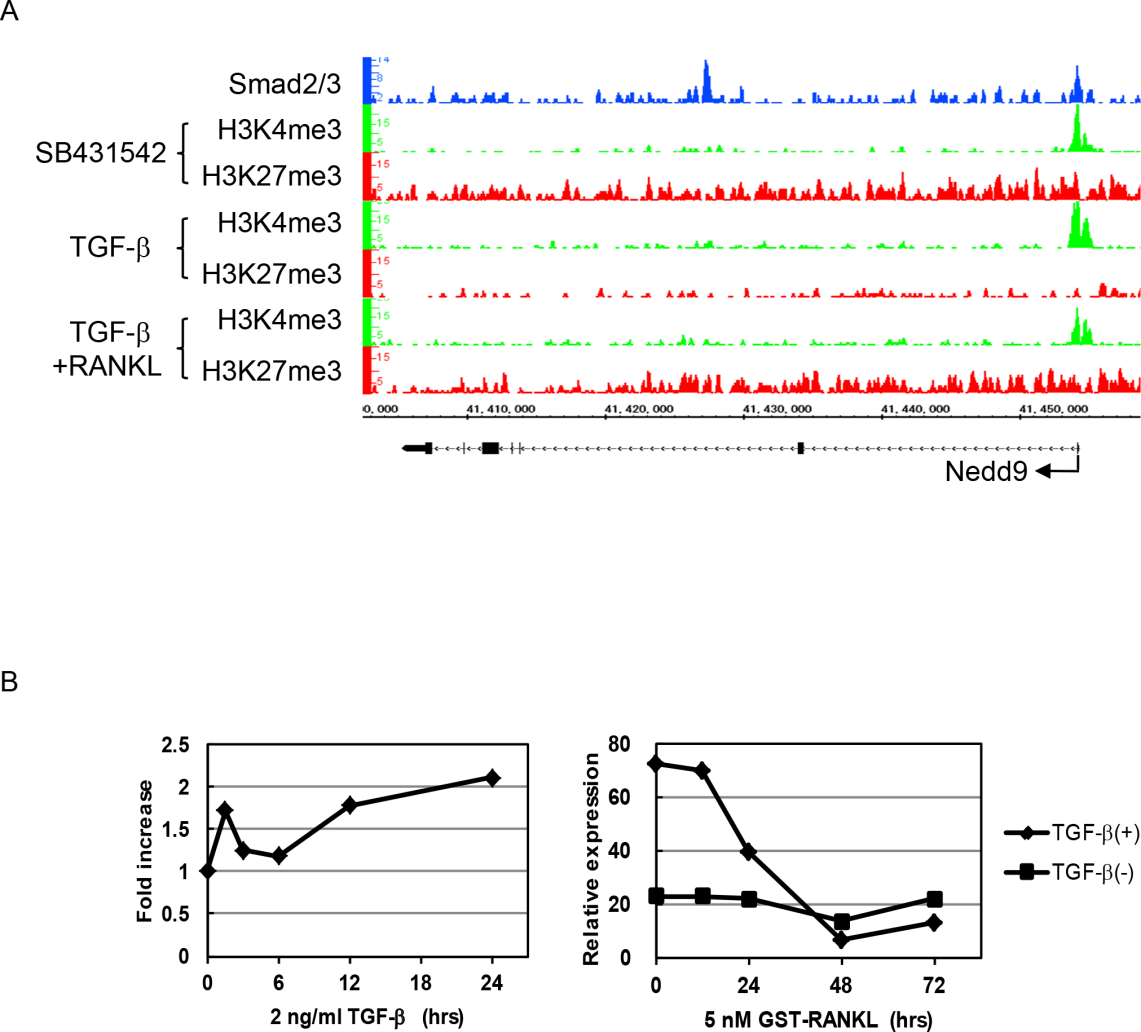
Epigenetic regulation of Nedd9 gene during osteoclastogenesis. (A) Smad2/3 binding and histone modification changes of *Nedd9* gene during osteoclastogenesis. Histone modification patterns in BMMs changed from K4(+)K27(+) to K4(+)K27(-) patterns by TGF-ß and returned to K4(+)K27(+) patterns after RANKL treatment. (B) *Nedd9* mRNA expression after TGF-ß or RANKL stimulation. The expression increased by TGF-ß stimulation and was reduced after RANKL treatment. The expression remained at low levels in the presence of SB431542.

We identified 14 Smad2/3 target genes in which histone modification patterns changed from K4(+)K27(+) to K4(+)K27(-) by TGF-ß treatment (Fig 5A). Nedd9, also called Cas-L or HEF-1 (human enhancer of filamentation 1), is a member of the CAS (crk-associated substrate) family proteins [18] [19] [20]. Nedd9 is a scaffold protein localized in focal adhesion that is involved in the development and progression of cancer cells [21] [22] [23]. As shown in Fig 3A, Smad2/3 binding was enriched around the TSS of *Nedd9* gene. Histone modification status changed from K4(+)K27(+) to K4(+)K27(-) patterns by TGF-ß stimulation in BMMs and then returned to K4(+)K27(+) patterns in response to RANKL stimulation. In fact, *Nedd9* gene expression was upregulated by TGF-ß in BMMs and downregulated by RANKL stimulation (Fig 5B).

**Fig 6:**
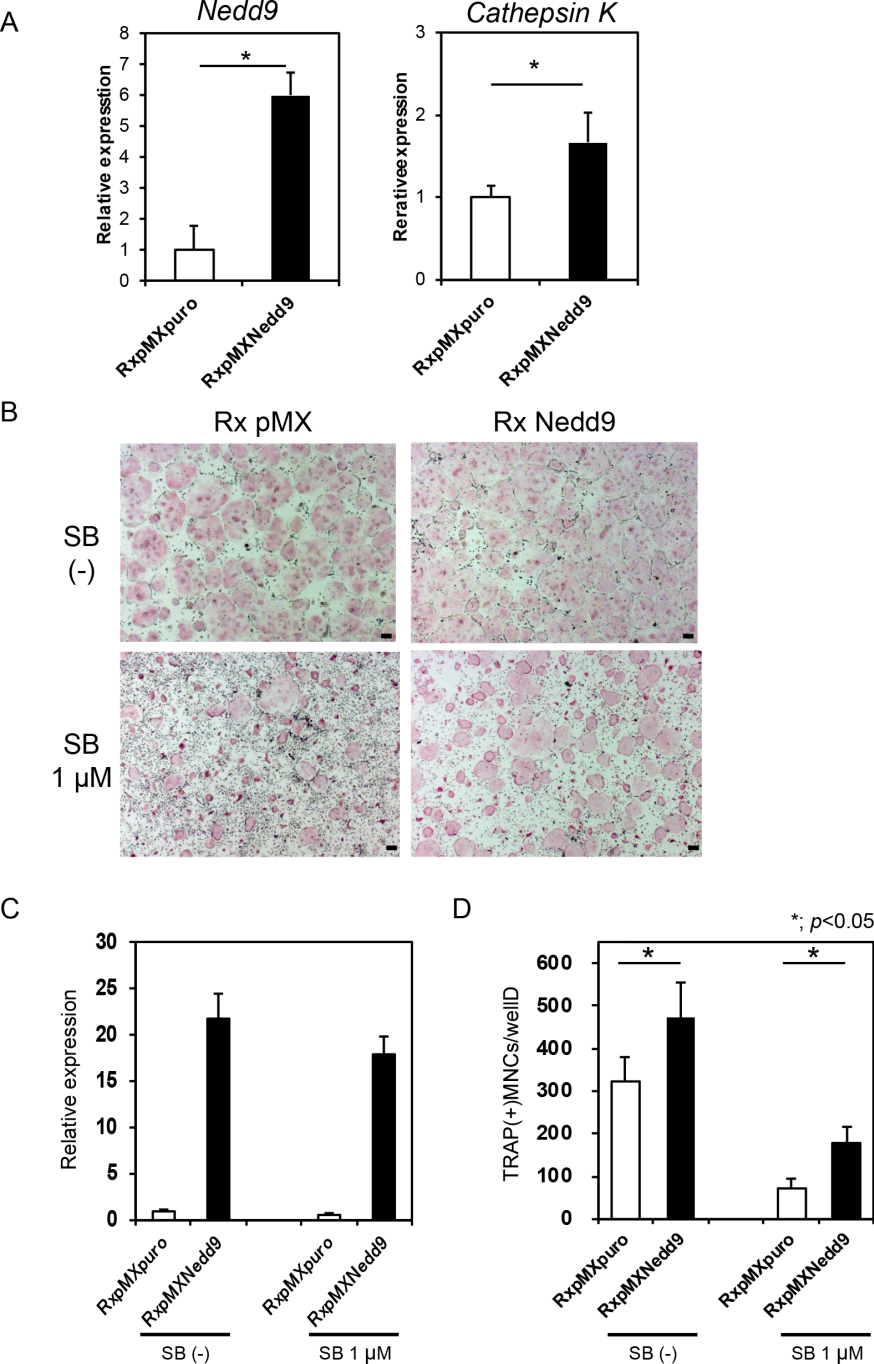
Nedd9 is critical for osteoclast differentiation. (A) Realtime PCR analysis of effects of retroviral overexpression of *Nedd9* gene on RANKL-induced osteoclastogenesis, as indicated by expression of *Nedd9* and *Cathepsin K* mRNAs. (B) Effects of SB431542 (SB) treatment on osteoclastogenesis, as evaluated by TRAP staining, in *Nedd9*-overexpressing cells treated with RANKL. Overexpression of *Nedd9* increased RANKL-induced osteoclastogenesis. SB431542 suppressed osteoclastogenesis, which was partly recovered by *Nedd9* overexpression. Cultures were stained by TRAP. Bars = 100 μm. (C) The expression of *Nedd9* gene as determined by realtime PCR. (D) The number of TRAP positive osteoclasts was significantly increased by *Nedd9* overexpression, and the suppression of osteoclastogenesis by SB431542 was partly suppressed by *Nedd9* overexpression. *P < 0.05.

**Fig 7:**
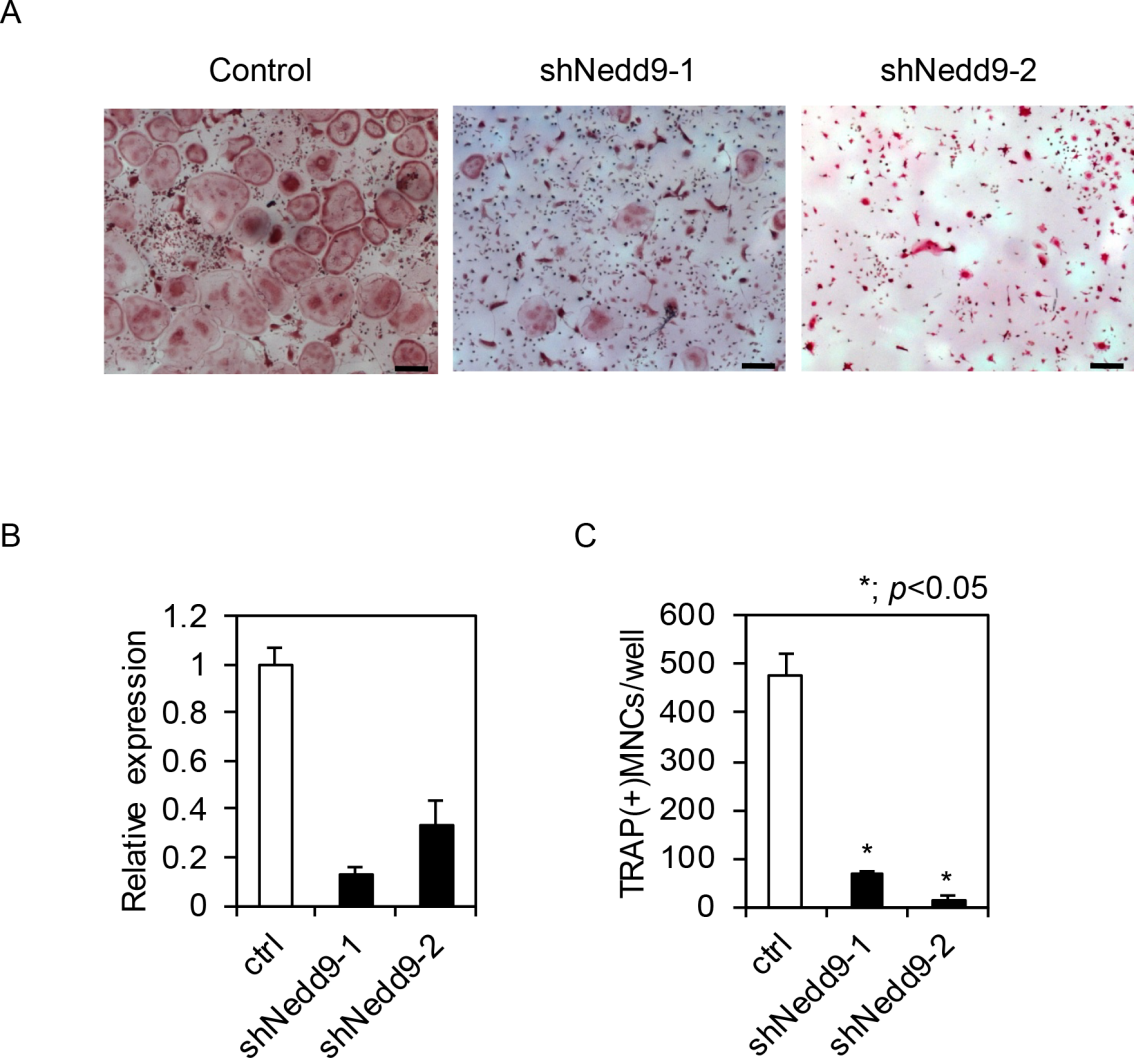
(A) Effects of knockdown of *Nedd9* gene by shRNA on osteoclastogenesis. BMMs infected with retrovirus vector carrying *shNedd9* were treated with 5 nM RANKL for 3 days and stained with TRAP. (B) Expression of *Nedd9* gene by realtime PCR. (C) The number of TRAP positive osteoclasts was significantly reduced by retroviral introduction of *shNedd9*. Bars = 100 μm. *P < 0.05.

We then analyzed a potential function for Nedd9 in osteoclastogenesis. Retroviral overexpression of *Nedd9* significantly increased the expression of *Cathepsin K*, a marker gene of osteoclasts (Fig 6A). In addition, *Nedd9* overexpression partially rescued the inhibitory effect of SB431542 on RANKL-induced osteoclastogenesis (*P* < 0.05; Fig 6B–6D). Conversely, *Nedd9* knockdown by retrovirus carrying *shNedd9* markedly suppressed RANKL-induced osteoclastogenesis (Fig 7A–7C). All of these findings strongly suggest a critical function for Nedd9 in RANKL-induced osteoclastogenesis.

**Fig 8:**
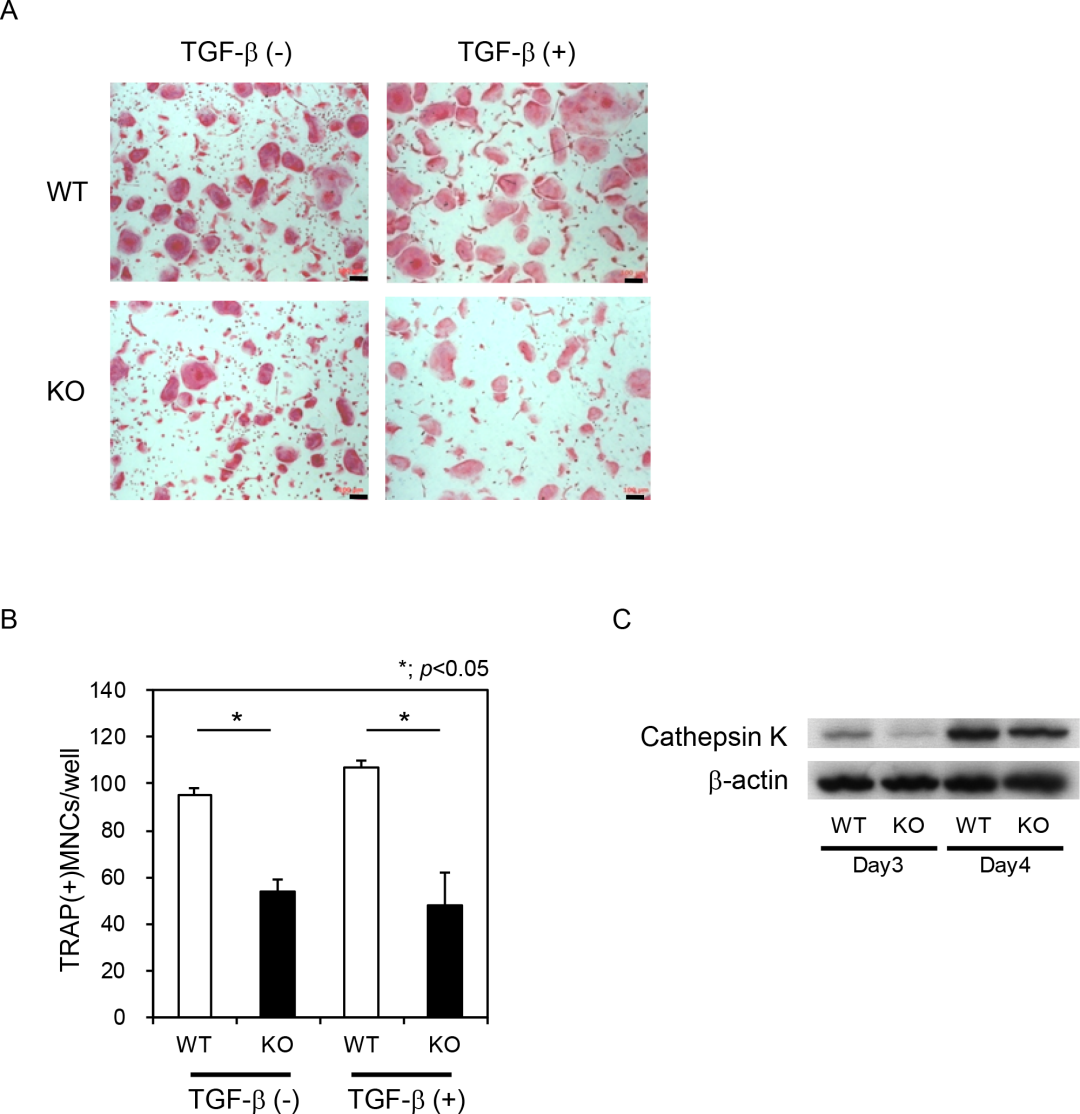
Impaired osteoclastogenesis in Nedd9-/- BMMs. (A) BMMs were isolated from *Nedd9* knockout (KO) and wild-type (WT) mice and cultured in the presence of RANKL with or without TGF-ß. Osteoclastogenesis was evaluated by TRAP staining (A), the number of multi-nuclear osteoclasts (B) and the expression of Cathepsin K protein (C).

**Fig 9:**
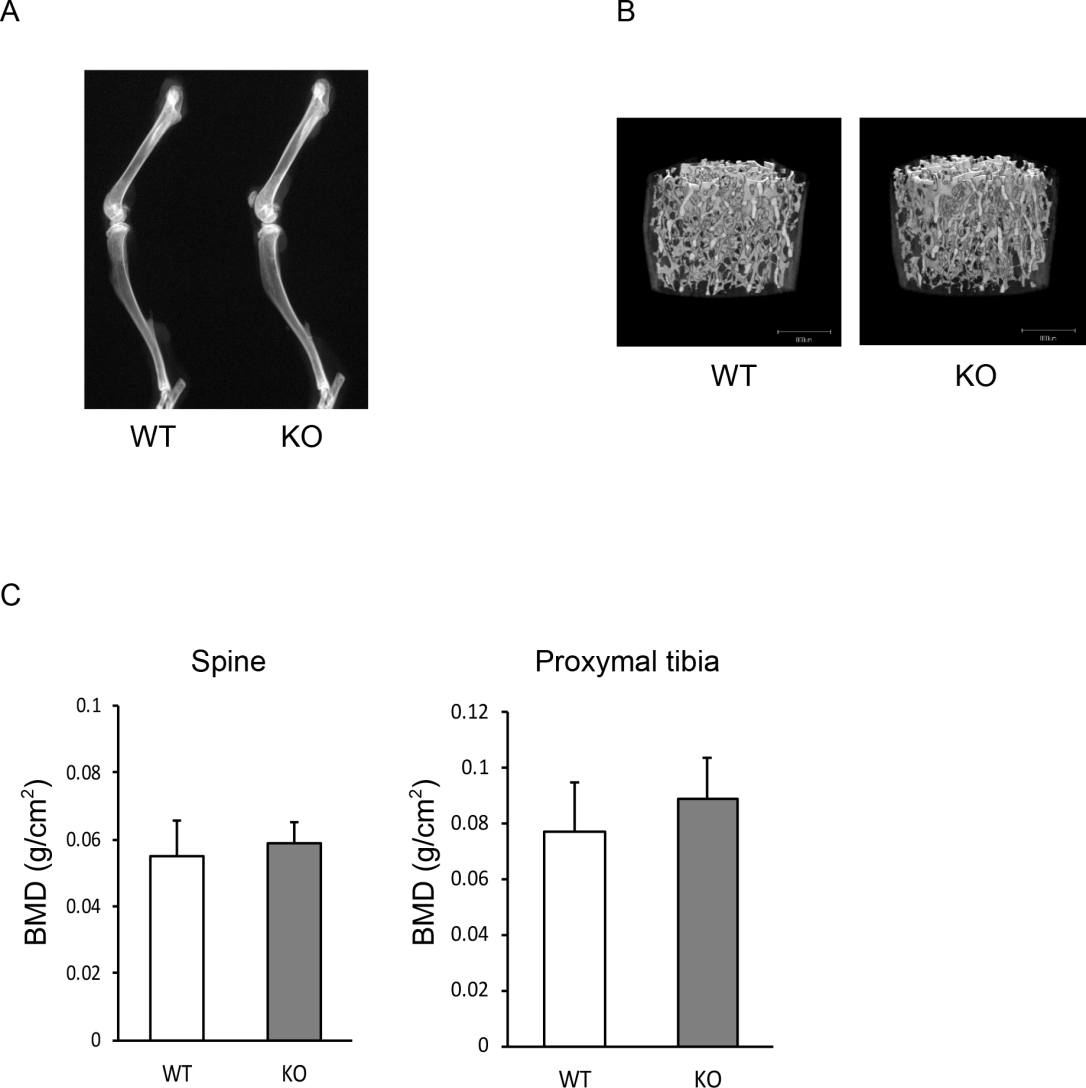
(A) Bone phenotypes of 12-week-old male *Nedd9*-/- and wild-type mice were evaluated using soft-X ray of the lower extremities. (B) micro CT in lumbar vertebral bodies (C) DEXA in lumbar vertebral bodies and proximal tibia.

Finally, we assessed the function of Nedd9 in osteoclastogenesis using *Nedd9*-/- mice. BMMs from *Nedd9*-/- mice exhibited reduced osteoclastogenesis similar to *shNedd9*-treated cells (Fig 8A and 8B). Stimulatory effects of TGF-ß on RANKL-induced osteoclastogenesis observed in wild-type BMMs was reduced in *Nedd9*-deficient BMMs (Fig 8B). Expression of Cathepsin K, as determined by Western blotting, was downregulated in *Nedd9*-deficient osteoclasts as compared to wild-type osteoclasts (Fig 8C). However, no significant difference in the skeletal phenotypes, as assessed by soft X ray in the lower extremities, micro CT in lumbar vertebral bodies, and dual energy X ray absorptiometry was observed between *Nedd9*-knockout and wild-type mice (Fig 9A–9C).

## Discussion

We previously reported that TGF-ß is indispensable for RANKL and M-CSF-induced osteoclastogenesis. However, the effector genes acting downstream of TGF-ß-Smad2/3 pathways remain elusive. In the present study, we comprehensively analyzed Smad2/3-binding regions and identified Smad2/3 target genes in BMMs by ChIP-seq analysis. Smad2/3 target genes were enriched in TGF-ß upregulated genes as expected. Interestingly, these genes were enriched in the gene population whose expression was downregulated by RANKL treatment, indicating the possibility that the role of TGF-ß is to prepare an appropriate condition for BMMs to differentiate into mature osteoclasts upon RANKL stimulation.

Histone modifications play important roles in cell differentiation. H3K4me3 is enriched in the active and poised promoter regions [24, 25], while H3K27me3 is involved in polycomb-mediated gene repression [26]. Recent studies have revealed that key developmental genes tend to change histone modification patterns from the H3K4me3/H3K27me3 bivalent pattern to the H3K4me3 monovalent pattern in various types of cells. In an attempt to narrow down the candidates of Smad2/3 target genes involving in osteoclast differentiation, we identified *Nedd9* as a Smad2/3 target gene whose histone modification pattern changed from K4(+)K27(+) to K4(+)K27(-) patterns in response to TGF-ß. Nedd9, also known as CasL and HEF1, was originally identified as a 105 kDa protein that is tyrosine phosphorylated by the ligation of ß1 integrins in peripheral T cells [18]. Nedd9 is induced by TGF-ß and directly interacts with Smads in various types of cells [27] [28]. We found that Smad2/3 binds to the promoter region of *Nedd9* gene and TGF-ß upregulates *Nedd9* expression in BMMs. Overexpression of *Nedd9* promoted and knockdown or knockout of *Nedd9* suppressed osteoclastogenesis, indicating a role of Nedd9 downstream of RANKL-RANK pathways. Previous studies reported that Nedd9 is involved in tumor differentiation, migration and metastasis [29], and therefore, it is also possible that Nedd9 supports osteoclast motility as well.

A previous study reported the association between Nedd9 and Smad6 or Smad7 [30], and our ChIP-seq analysis showed that Smad2/3 bound to the promoter region of *Smad6* and *Smad7*. Although we did not address the association between inhibitory Smads and Nedd9 in BMMs, it is possible that Nedd9 regulates TGF-ß signaling in a negative feedback manner by interacting with inhibitory Smads.

The role of Nedd9 in skeletal homeostasis *in vivo* is not clear. Seo et al. generated *Nedd9*-deficient mice and reported that lymphocyte trafficking was altered [22]. In addition, Katayose et al. recently reported that *Nedd9*-/- mice exhibited decreased onset of collagen-induced arthritis compared with wild-type mice and that joint destruction was reduced in the knockout mice [31]. We found that RANKL-induced osteoclastogenesis was impaired in BMMs from *Nedd9*-/- mice, and the stimulatory effect of TGF-ß on RANKL-induced osteoclastogenesis was partially abrogated. However, we were unable to find the abnormal bone phenotypes in *Nedd9*-/- mice. This may be due to the effect of Nedd9 on other types of cells such as osteoblasts or osteocytes, or the effect of Nedd9 deficiency is only observed in mice under pathological conditions such as ovariectomy and arthritis, or those treated by TGF-ß. Further studies are required to fully understand the role of Nedd9 in the skeletal milieu.

## Materials and Methods

### Reagents

Recombinant human M-CSF was purchased from R&D Systems (Minneapolis, MN, USA), and TGF-β and SB431542 were from Sigma-Aldrich (St Louis, MO, USA). GST-RANKL was purchased from Oriental Yeast Co., Ltd (Shiga, Japan). Alpha-minimum essential medium (α-MEM) and fetal bovine serum (FBS) were purchased from Life Technologies (Carlsbad, CA, USA). Smad2/3 antibody was purchased from BD Biosciences (Monoclonal antibody, Mouse, 610843, San Jose, CA, USA), anti-trimethyl-histone H3 lysine 4 was from Activemotif (Polyclonal antibody, Rabbit, 39159, Carlsbad, CA, USA), anti-trimethyl-histone H3 lysine 27 was from Millipore (Polyclonal antibody, Rabbit, 07–449, Billerica, MA, USA), anti-β-actin was from Sigma-Aldrich (Polyclonal antibody, Rabbit, A2066, St Louis, MO, USA).

### Animals

*Nedd9*-/- mice with C57BL/6J (B6) genetic background were generated as previously reported [22]. In short, the exon 2 encoding the N-terminal SH3 domain in the Cas-L protein was replaced with EGFP and a neomycin resistance gene. All animal procedures were approved by the Animal Care and Use Committee of the University of Tokyo.

### Cell Culture

Murine bone marrow cells were collected from the femur and tibia of male ddY mice at 4–5 weeks of age. To prepare BMMs, cells were cultured in α-MEM/10% FBS with 100 ng/ml M-CSF for 5 days. BMMs were further cultured in the presence of 10 ng/ml M-CSF and 100 ng/ml RANKL for 4 days to generate osteoclasts. To examine the effect of the TGF-β-Smad pathway on osteoclastogenesis, 1 ng/ml TGF-β or 10 μM SB431542 was added with RANKL. Cells were stained with tartrate-resistant acid phosphatase (TRAP), and TRAP-positive cells containing more than three nuclei were counted as osteoclasts32.

### Real-time PCR analysis

Total RNA was extracted with ISOGEN (Wako Pure Chemical Industries, Ltd.), and a 1-μg aliquot was reverse transcribed using a QuantiTect Reverse Transcription kit (QIAGEN) to produce singlestranded cDNA. PCR was performed on an ABI Prism 7000 Sequence Detection System (Applied Biosystems) using QuantiTect SYBR Green

PCR Master Mix (QIAGEN) according to the manufacturer’s instructions. All reactions were performed in triplicate. After data collection, the mRNA copy number of a specific gene was calculated with a standard curve generated with serially diluted plasmids containing PCR amplicon sequences and then normalized to rodent total RNA with mouse ß-actin serving as an internal control. Primer sequences were as follows: Nedd9 forward, 5′-CCACCCTCCTACCAGAATCA-3′; Nedd9 reverse, 5′-ATACCCCTTGAGTGCTGTGG-3′; *Cathepsin K*-forward, 5’-ACGGAGGCATTGACTCTGAAGATG-3’; *Cathepsin K*-reverse, 5’-GGAAGCACCAACGAGAGGAGAAAT-3’.

### Expression constructs and gene transduction

For retrovirus construction, the full-length cDNAs were amplified by PCR using KODplus (Takara Bio Inc.), subcloned into Zero Blunt TOPO II vectors (Invitrogen), and inserted into pMX-puro vectors. A total of 2 × 10^6^ BOSC23 packaging cells were transfected with 6 μg of vector using FuGENE 6 (Roche). After 24 h, the medium was replaced with fresh α-MEM/10% FBS, and cells were incubated for an additional 24 h. The supernatant was then collected as retroviral stock after centrifugation at 2,400 rpm for 3 min. A total of 5×10^6^ BMMs were incubated with 8 ml of retroviral stock for 5 h in the presence of 6 μg/ml polybrene and 30 ng/ml recombinant mouse M-CSF. After 5 h of retroviral infection, the medium was changed to α-MEM/10% FBS and 100 ng/ml M-CSF, and cells were cultured for an additional 24 h. BMMs were recovered with trypsin, and puromycin-resistant cells were selected by incubation with α-MEM/10% FBS containing 2 μg/ml puromycin for 2 d and used for further experiments.

### RNA interference (RNAi)

RNAi expression vectors were constructed with piGENEmU6 vector (iGENE Therapeutics; Tokyo, Japan) as described [32] [33], and the U6 promoter and inserts were cloned into pMx vectors. Retroviruses carrying specific genes were prepared by transfecting BOSC packaging cells with retrovirus vectors and collecting the supernatant after 2 days. For retroviral infection, after the first 2 days of culture, BMMs were incubated with retrovirus in the presence of 30 ng/ml M-CSF and 4 ng/ml polybrene (Sigma-Aldrich; St Louis, MO, USA) for 6 h and cultured overnight in the presence of 10 ng/ml M-CSF. To select the transduced BMMs, cells were detached with Trypsin/EDTA (Sigma-Aldrich; St Louis, MO, USA) and cultured with 10 ng/ml M-CSF and 2 μg/ml puromycin (Sigma-Aldrich) for 2 days. Primer sequences were as follows: shNedd9_1 (sense) 5′-gtttGCATTAGATCTTTGGTCGACAgtgtgctgtccTGTTGGCCAAAGGTCTGATGCttttt-3′, shNedd9_1 (antisense) 5′-atgcaaaaaGCATCAGACCTTTGGCCAACAggacagcacacTGTCGACCAAAGATCTAATGC-3′, shNedd9_2(sense) 5′-gtttGCAGTGCTAGGAGTGACATGTgtgtgctgtccACATGTTACTCCTGGTACTGCttttt-3′, shNedd9_2 (antisense) 5′-atgcaaaaaGCAGTACCAGGAGTAACATGTggacagcacacACATGTCACTCCTAGCACTGC-3′.

### ChIP and ChIP-seq

Cells were cultured in 15-cm plates to approximately 80% confluency. Cells were fixed with 1% formaldehyde at room temperature and then neutralized with glycine. After cells were collected and re-suspended, samples were sonicated. Samples were incubated with protein A/G beads that had been pre-incubated with 4–10 μg of antibody. Immunoprecipitates were washed and reverse-crosslinked, and DNA was purified with a PCR purification kit (Qiagen, Germantown, MD, USA). DNA libraries were prepared for sequencing using the standard Illumina protocol. Purified DNA was applied for cluster generation and sequencing on the cBot Cluster Generation system and Genome Analyzer IIx system (Illumina; San Diego, CA, USA) following the manufacturer’s instructions. Obtained sequences were mapped to the reference mouse genome. ChIP primer sequences were as follows: Nedd9 forward: 5′-AGAAGGCAGAGGCAGCATAA-3′, Nedd9 reverse: 5′-CCTGTGGCATCATCTCTAAGG-3′, Pdgfb forward: 5′- TTTCAAGGCGATGAGGTCAC-3′, Pdgfb reverse: 5′- GGAGAGTGCCCCAGACCT -3′, Smad6　forward: 5′-CATGCAGGGTGTCTCTAGCA-3′, Smad6　reverse: 5′-GGCTACATGGATCACGATGG-3′, Tmepai forward: 5′-AAACCTACTGCGACGACAGG-3′, Tmepai reverse: 5′-ATGAGAGGCACTTTGCAACC-3′, Ski forward: 5′-TGGAGAGGCTCTGCTCTAGG-3′, Ski reverse: 5′-CCTGCAGCTGGTTTGTGTAA-3′, Cdkn1a forward: 5′-TCTGT GTACGTGCGTGTGTG-3′, Cdkn1a reverse: 5′-TAAATTCCCGCCTATGTTGG-3′, Serpine1 forward: 5′-AGCCCAATAGAGAACTTCAAGTCC-3′, Serpine1 reverse: 5′-CAGTACACCTCAAAACCCAGCC-3′, Smad7 forward: 5′-TGCGAAACACAATCGCTTT-3′, Smad7 reverse: 5′-CTCTGCTCGGCTGGTTCC-3′, Ppib forward: 5′-ATGTGGTACGGAAGGTGGAGA-3′, Ppib reverse:　5′-AGCTGCTTAGAGGGATGAGG-3′, Hoxa13 forward: 5′-TGGCATGTTTTAGGGACCTC-3′, Hoxa13 reverse: 5′-CACATCCTTGGGAGGGTCTA-3.

### Microarray expression array analysis

Total RNA was extracted with TRIzol and subjected to GeneChip (Affymetrix; Santa Clara, CA, USA) expression analysis according to the technical manual. Briefly, biotin-labeled cRNA synthesized from total RNA was hybridized to a GeneChip Mouse Genome MOE430 2.0 oligonucleotide array (Affymetrix; Santa Clara, CA, USA). The arrays were stained with streptavidin-phycoerythrin and image data was collected with an Affymetrix scanner. Microarray Suite software 5.0 was used to calculate the average difference (AD) for each gene probe set, shown as the gene expression intensity value. The AD values were normalized for each array so that the average of all AD values was 100. One array datum was obtained for each sample. Obtained data were verified by qPCRs for various transcripts, and we had no conflicting results between the array data and qPCR data. Affymetrix probe IDs were converted to gene symbols.

### Statistical analyses

The results are expressed as mean±SD. Statistical analyses were performed using a two-tailed unpaired Student's *t* test for continuous variables and chi-square tests for categorical variables. A p value of less than 0.05 was considered to be statistically significant.
